# Noncanonical Functions and Cellular Dynamics of the Mammalian Signal Recognition Particle Components

**DOI:** 10.3389/fmolb.2021.679584

**Published:** 2021-05-25

**Authors:** Camilla Faoro, Sandro F. Ataide

**Affiliations:** School of Life and Environmental Sciences, The University of Sydney, Sydney, NSW, Australia

**Keywords:** signal recognition particle, ribonucleoproteins, RNA-binding proteins, co-translational targeting, noncanonical functions

## Abstract

The signal recognition particle (SRP) is a ribonucleoprotein complex fundamental for co-translational delivery of proteins to their proper membrane localization and secretory pathways. Literature of the past two decades has suggested new roles for individual SRP components, 7SL RNA and proteins SRP9, SRP14, SRP19, SRP54, SRP68 and SRP72, outside the SRP cycle. These noncanonical functions interconnect SRP with a multitude of cellular and molecular pathways, including virus-host interactions, stress response, transcriptional regulation and modulation of apoptosis in autoimmune diseases. Uncovered novel properties of the SRP components present a new perspective for the mammalian SRP as a biological modulator of multiple cellular processes. As a consequence of these findings, SRP components have been correlated with a growing list of diseases, such as cancer progression, myopathies and bone marrow genetic diseases, suggesting a potential for development of SRP-target therapies of each individual component. For the first time, here we present the current knowledge on the SRP noncanonical functions and raise the need of a deeper understanding of the molecular interactions between SRP and accessory cellular components. We examine diseases associated with SRP components and discuss the development and feasibility of therapeutics targeting individual SRP noncanonical functions.

## Introduction

The precise cellular localization of nascent proteins is an essential process required for maintaining homeostasis, cell organization and survival (Hartl et al., 2011). Proteins can be delivered to their correct compartment during (co-translational) or after (post-translational) their biosynthesis in the cytoplasm. Roughly 30% of the proteome is initially destined to the endoplasmic reticulum (ER) in eukaryotes or the plasma membrane in bacteria. The main pathway for proteins to enter the ER is *via* co-translational translocation. Co-translational protein export is an efficient process that exquisitely interconnects protein translation with cell compartmentalization to circumvent challenges in folding and processing that newly nascent polypeptide may face if released into the cytoplasm (Hegde and Keenan, 2011). Given the complexity of these tasks, the cell has evolved specialized machines to achieve this goal (Cross et al., 2009).

The co-translational pathway utilizes the Signal Recognition Particle (SRP), an essential molecular machinery that couples the synthesis of nascent proteins to their proper secretory pathway and membrane localization (Akopian et al., 2013). Since the postulation of the “signal hypothesis” in 1971 and its discovery in 1980 by Walter and Blobel, SRP has been extensively investigated across diverse research fields focusing on its structure and molecular translocation function (Akopian et al., 2013). However, in the last 20 years, multiple studies have identified the SRP components in new interactions outside the co-translational translocation machineries, suggesting new roles for its components in regulating multiple pathways.

Noncanonical functions associated with RNA-binding proteins (RBPs) and ribonucleoprotein complexes (RNPs) are not unusual and ever since their discoveries, several studies have uncovered a new universe of RNA-biding activities. For instance, telomerase has been associated with extratelomeric properties that are independent of its role in telomere extension, implicating selective-telomerase targeted cancer therapies (Li and Tergaonkar, 2014). Cajal body-specific RNPs (scaRNPs), which play a role in the biogenesis of small nuclear RNPs (snRNPs), have been proposed to act as regulatory RNPs (regRNPs) that contribute to ribosome heterogeneity (Poole et al., 2017).

The role of SRP components in cellular events outside co-translation translocation, such as cell growth, differentiation and death, remain largely unexplored and underappreciated in mammalian cell biology. This review will discuss for the first time the emerging noncanonical roles of SRP components in mammalian cells reported to date, along with their association with cancer, autoimmune and genetic diseases.

## Canonical SRP Pathway

### The SRP Cycle and Its Composition

**FIGURE 1:**
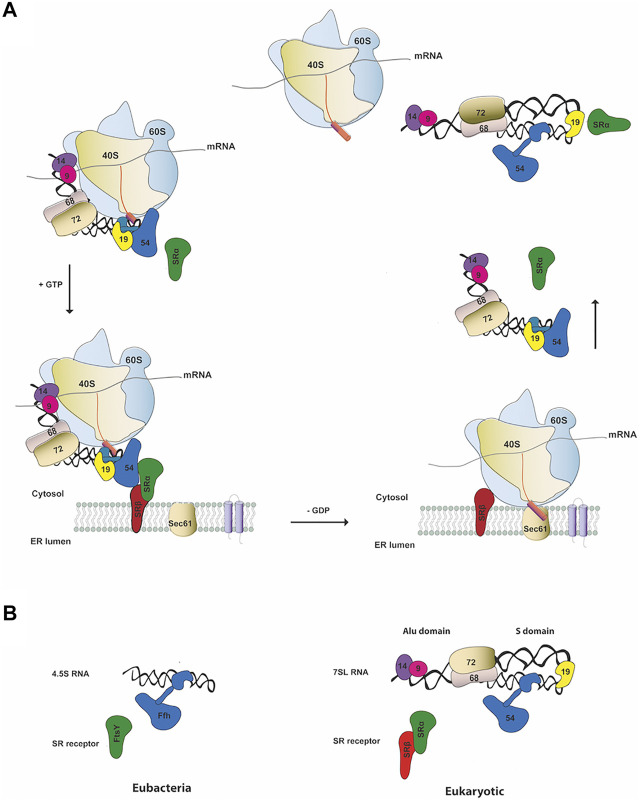
SRP canonical cycle and composition **(A)** Schematic representation of co-translation protein targeting pathway in mammals. SRP binds the signal sequence (pink cylinder) as it emerges from the ribosome forming an RNC-SRP complex that docks in a GTP-dependent manner with the ER membrane by binding to the cognate SRP receptor (SR α/β). Following GTP hydrolysis, the RNC is transferred to the Sec61 translocon resulting in translocation of the nascent chain through the Sec61 pore and disassemble of SRP-SR complex **(B)** Schematic representation of SRP and SR components in the two kingdoms of life. On the left panel it is shown the eubacterial SRP complex and on the right the eukaryotic one (e.g., human SRP).

The canonical role of SRP involves co-translational targeting of secretory proteins to the ER in eukaryotes or to the plasma membrane in prokaryotes. The SRP-dependent protein pathway involves a series of sequentially regulated steps (Figure 1A). Proteins destined to enter the secretory pathway typically possess N-terminal hydrophobic signal sequences, which direct them to their target membrane (Rapoport, 2007). During the SRP cycle, SRP recognizes the hydrophobic signal peptide emerging from the exit tunnel of the translating ribosome and forms a ribosome nascent chain (RNC)–SRP complex (Pool et al., 2002; Halic et al., 2006). In eukaryotes, recognition of the signal sequence by SRP leads to arrest of the elongation of the polypeptide chain (elongation arrest) (Walter and Blobel, 1981). The RNC–SRP complex then docks in a GTP-dependent manner with its cognate SRP receptor (SR) on the target membrane. The signal peptide is released from SRP and the RNC is transferred to the translocon machinery (SecYEG in prokaryotes and Sec61p in eukaryotes). Meanwhile, translation resumes and the newly synthesized protein is delivered into the ER or plasma membrane (Saraogi and Shan, 2011). GTP hydrolysis by SRP:SR causes their dissociation and recycle for further rounds of targeting (Connolly et al., 1991).

SRP is a universally conserved ribonucleoprotein complex (RNP) found in all domains of life that has evolved its composition and function to become more complex and sophisticated in eukaryotes (Pool, 2005). However, the central ribonucleoprotein core and the general mechanism of SRP targeting are highly conserved. Bacteria contains the simplest and smallest SRP, comprising the universally conserved SRP54 protein (called Ffh) and the shorter 4.5S RNA (Figure 1B, left panel). Mammalian SRP is the most complex particle and consists of a long noncoding RNA (lncRNA) of approximately 300 nucleotides, the 7SL RNA, and six protein subunits named according to their apparent molecular weight: SRP9, SRP14, SRP19, SRP54, SRP68, and SRP72 (Figure 1B, right panel) (Peter Walter, 1983). The mammalian SR is a heterodimer composed of two subunits, SRα and SRβ. SRα is a 69 kDa peripheral membrane protein while SRβ is a 25 kDa integral ER membrane protein (Tajima et al., 1986). SRα regulates the SRP targeting cycle by interacting with SRP54 and with the translocon, while SRβ is responsible to target the SRP at the ER membrane. Notably, SRα is universally conserved while SRβ subunit is exclusive to eukaryotes. FtsY (Filamentation temperature sensitive X), the SR bacterial receptor, is the functional homologue of the mammalian SRα (Miller et al., 1994).

The eukaryotic SRP can be separated into two structural and functional domains, the Alu and S domains (Figure 1B, right panel) (Gundelfinger et al., 1983). The Alu-domain is formed by the 5′-3′ end of the SRP 7SL RNA, SRP9 and SRP14 subunits and is responsible for arresting translation elongation (Weichenrieder et al., 2000). The S-domain is responsible for protein translocation activities by recognition of the signal sequence and for GTP-dependent interaction with the receptor and comprises the central region of the SRP 7SL RNA, proteins SRP19, SRP54, SRP68 and SRP72 (Walter and Blobel, 1983).

### Structural Highlights of the SRP Components

7SL RNA has an architectural and enzymatic role in SRP assembly and acts as a central regulator of the SRP function, mediating global reorganization of the SRP in response to cargo binding and molecular communication with the cognate receptor. The 7SL SRP RNA folds into a double-stranded secondary structure with a cruciform shape where the Y-shaped S domain constitutes the central region of the RNA. From a structural point of view, it can be divided in twelve helices (1–12), four domains (I-IV) and four conserved motifs that represent the interaction sites for SRP proteins (Zwieb, 1985; Zwieb et al., 2005).

SRP9 and SRP14 possess an αβββα fold with topology similar to double strands RBPs, consisting of a three-stranded antiparallel β-sheet stacked against two α-helices (Birse et al., 1997). They form a stable and obligate heterodimer that recognizes the RNA UGUNR motif, localized in the highly conserved 5’ end of the Alu domain of the 7SL RNA, called the τ-junction, which fold compactly as two helical stacks linked by U-turn (Weichenrieder et al., 2000). Consistent with its role in elongation arrest, the Alu domain binds in the ribosomal subunit interface (Halic et al., 2004).

SRP19 plays an essential role in the assembly of eukaryotic SRP. It is a single domain protein that belongs to the αβ folding class of RBPs with a βαββα topology (Wild et al., 2001). The most basic region of SRP19 binds specifically to the tip of the GGAG tetraloop of helix 6 of the SRP RNA (Oubridge et al., 2002). This interaction clamps helices 6 and 8 together, inducing the typical closed S domain structure.

SRP54 is a key component in SRP signal sequence recognition and interaction with the ribosome and with the SRP receptor (Connolly and Gilmore, 1989). The protein contains three domains, named N, G and M. The N and G domains form a functional unit called NG domain that confers GTPase activity during the SRP cycle and mediates SRP-SR interaction (Freymann et al., 1997). The hydrophobic C-terminal methionine-rich M domain has a helical fold and provides the RNA- and the signal sequence-binding sites (Keenan et al., 1998; Batey et al., 2000). 

SRP68 and SRP72 are the least characterized SRP proteins, in terms of structure and function. SRP68 and SRP72 form a stable heterodimer that is essential for SRP functions and is suggested to coordinate the action of the S and Alu domain, facilitating elongation arrest after signal sequence recognition (Siegel and Walter, 1988; Andreazzoli and Gerbi, 1991). These two largest SRP components bind to the central region of the 7SL SRP RNA, around the three way junction formed by helix 5, 6, 7, and 8, and both consist of a RNA-binding domain (RBD) and a protein-binding domain (PBD) (Yin et al., 2007). SRP68 has a quite large RNA-binding domain located at the N-terminus (52–252 aa) and contains a glycine-rich region typical of other RBPs, however it is not required for RNA binding (Herz et al., 1990). The relatively small RNA-binding region of SRP72 shares a conserved Pfam motif and a cluster of positively charged amino acids (Iakhiaeva et al., 2010). SRP72 protein binding domain is composed of nine antiparallel α-helices arranged in a closed α-solenoid. The domain comprises four tetratricopeptide repeats (TPRs) that binds the SRP68 protein binding domain, located at its C-terminus, in a conserved TPR-groove (Becker et al., 2017). SRP68-RBD (52–252 a. a) is a tetratricopeptide-like module composed of 7 antiparallel α-helices and an extended loop region. SRP68 binding bends the RNA S domain and inserts an α-helical arginine-rich motif (ARM) into the major groove (Grotwinkel et al., 2014).

A more recent cryo-EM structure (PDB 6FRK) describes the mammalian translating ribosome in complex with SRP and SR in a conformation preceding signal sequence delivery. The structure was determinated at 4.5 to 10 Å resolution and allows to observe the architecture of the entire mammalian SRP-SR-RNC complex (Kobayashi et al., 2018).

## Noncanonical Functions of SRP and Accessory Binding Partners

SRP components were first identified as integral part of the SRP machinery, however they have been implicated in regulation of many cellular processes, including gene expression, viral infection, apoptosis and stress response (Table 1).

**TABLE 1:** Noncanonical functions and binding partners of SRP components.

SRP component(s)	Main noncanonical function(s)	Accessory complexes and binding partners	Key references
7SL RNA	HIV-1 virus-packaging	Gag, APOBEC3F, APOBEC3G	Tian et al. (2007); Wang et al. (2007b); Wang et al. (2008a)
	Inhibition of HBsAg expression	EDEM1 (?)	Wang et al. (2014)
	Red blood cells metabolism	RBCs	Talhouarne and Gall (2018)
	Cell-cell communication	Extracellular vesicles	Nabet et al. (2017)
	Proliferative effect	3′UTR TP53 mRNA, HuR	Abdelmohsen et al. (2014)
SRP9/14	Regulator of translation	Alu RNPs, 40 S ribosome	Bovia et al. (1995); Hasler and Strub (2006)
	Stress response	SGs	Berger et al. (2014)
SRP9, SRP54	HIV-1 virus-host interaction	HIV-Rev	Naji et al. (2012)
SRP14	HIV-1 virus-host interaction	HIV-Gag	Engeland et al. (2014)
SRP54	Transcription factor	SLC6A3	Zhao et al. (2018)
SRP68/72	Transcription factor	H4	Li et al. (2012)
	Histone networks	H1	Kalashnikova et al. (2013); Zhang et al. (2016)
	Targeting of nucleolin (?)	Cell-surface nucleolin complex	Krust et al. (2011); Destouches et al. (2012)
	HIV-1 virus-host interaction	Staufen1-RNP	Milev et al. (2012)
SRP68	Piggyback recruitment (?)	CASC-3 EJC-RNP complex	Mabin et al. (2018)
	Stress response Piggyback recruitment (?)	TRAPPC2–SGs	Zappa et al. (2019)
SRP72	Apoptosis regulation	Caspases (?)	Utz et al. (1998)

SRP proteins and RNA have a dynamic spatiotemporal distribution in response to different cell signals. They switch between assembling into SRP complex and acting by themselves independently from the SRP upon interaction with a multitude of binding partners. The nature of SRP binding partners and their interaction dictates their additional biological functions (Table 1). An early study has identified the 7SL RNA in an RNP complex with one protein that was not part of the SRP complex (Avanesov, 1988). The protein was not further characterized, but it had a molecular weight of 80–85 kDa, which differs from the SRP proteins. More recently, 7SL RNA has been found in red blood cells (RBCs) and exosomes, reinforcing the hypothesis that 7SL RNA may be a dynamic component of new RNPs (Nabet et al., 2017; Talhouarne and Gall, 2018).

SRP9/14 can exist as free heterodimer localized predominantly in the cytoplasm in 20-fold excess over the SRP complex, or be part of different RNPs, suggesting additional functions outside the SRP cycle (Bovia et al., 1995).

Performing a dynamic choreography, SRP68/72 heterodimer is either involved in protein targeting associated with the SRP complex or involved in histone-binding activity, transcription regulation and potentially other chromatin-related functions. The binding of SRP68/72 to H4 was the first evidence for SRP transcriptional regulation functions (Li et al., 2012), since then a transcription factor role was reported also for SRP54, expanding the picture of SRP-gene regulation (Zhao et al., 2018).

### SRP and Virus-Host Interactions

SRP RNA was initially believed to serve as a scaffold for the six SRP proteins to bind onto it. Now, 7SL RNA is found to be a dynamic component of SRP involved in a wide repertoire of biological pathways, especially in virus-host responses (Elvekrog and Walter, 2015). To date 7SL RNA is the most abundant non-tRNA species found in all retroviruses whose RNA contents has been determined (Brameier et al., 2013; Eckwahl et al., 2016). Different studies showed that 7SL RNA is selectively encapsulated into retroviruses, including Moloney murine leukemia virus (MuLV) (Duesberg and Robinson, 1966)*,* Rous sarcoma virus (RSV) (Bishop et al., 1970), Feline infectious leukemia virus (FeLV) (Brian et al., 1975), Visna virus (Lin and Thormar, 1971), Equine infectious anemia virus (EIAV) (Cheevers et al., 1977) and HIV-1 (Onafuwa-Nuga et al., 2006).

Packaging of 7SL RNA has been extensively studied in HIV-1 where it is encapsulated in a 6-fold excess to the genomic viral RNA (T. Wang et al., 2007). The mechanism of selective packaging of 7SL RNA into HIV-1 virions was elucidated by Tian and Didierlaurent. The 7SL RNA is mainly associated with viral core structures and Gag protein *via* its RNA-binding nucleocapsid (NC) domain (Tian et al., 2007; Didierlaurent et al., 2011). The proposed model consists of different regions of Gag interacting with multiple domains of 7SL RNA suggesting that it may play a role in retroviral assembly by guiding Gag to sites of assembly or by facilitating Gag multimerization. During HIV-1 virus assembly, 7SL RNA interacts with two cytidine deaminases APOBEC3 (A3G and A3F) to enhance their packaging into HIV-1 virions, however, this finding remains controversial (Khan et al., 2007; Bach et al., 2008; Wang et al., 2008b). Future studies are needed to clarify the nature and function of the interaction between 7SL RNA and A3G/A3F, with particular emphasis on their ability for precise HIV-1 targeting and viral inhibition.

**FIGURE 2:**
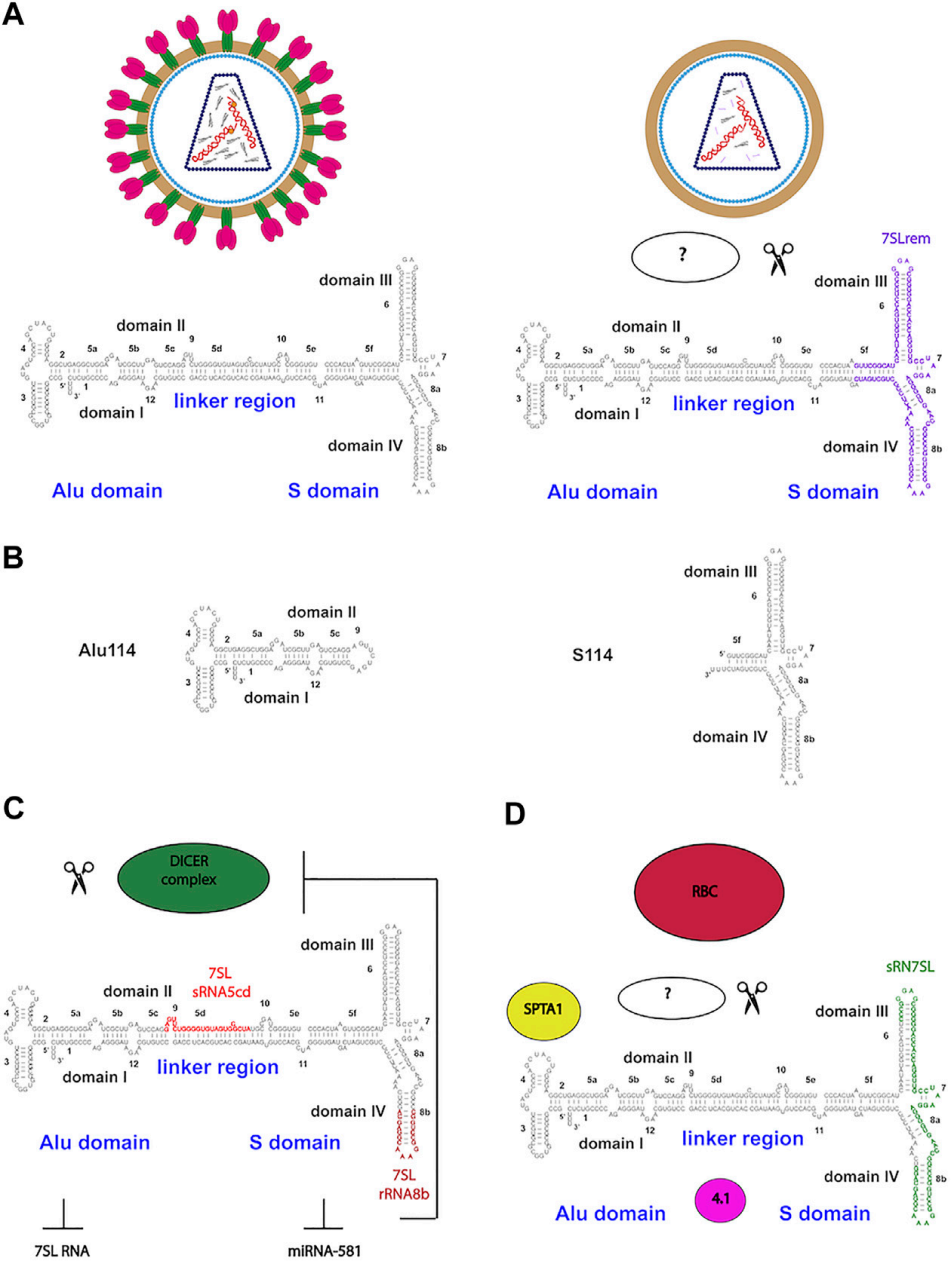
Functional endonuclease processing of 7SL RNA in HIV-1 particles and RBCs **(A)** *Trans*-acting packaging determinants of 7SL RNA during HIV-1 packing. HIV-1 mature and virus-like particles (VLPs) retained intact 7SL RNA in NC-positive VPLs (Gag-NC domain is shown as orange dots). In NC-negative VPLs, 7SL RNA is retained mainly as an endoribonucleolytic fragment of 111 nt, named 7SL remnant (7SLrem) **(B)** The *cis*-acting determinants of 7SL packaging by HIV-1. Schematics of the Alu (Alu114) and the S domain (S114) derivatives capable of mediating packaging of 7SL RNA **(C)** 7SL RNA processing by Dicer in fragments sRNA5cd and sRNA8b which repress SRP complex formation and the expression of HBV surface antigen (HBsAg) **(D)** Enrichment for 7SL RNA and its fragment sRN7SL in an RBC RNP containing SPTA1 and protein 4.1.

*Trans*-acting packaging factors have also been identified to interact with 7SL RNA during HIV-1 packing (Keene et al., 2010)*.* HIV-1 virus-like particles (VLPs) retained 7SL RNA mainly as an endoribonucleolytic fragment of 111 nt, named 7SL remnant (7SLrem) (Figure 2A). The presence of 7SLrem correlated with the absence of the NC domain of Gag in VLPs while intact 7SL RNA was present in NC-positive VPLs, indicating that the NC domain may protect 7SL RNA from processing and degradation. The 5′- and 3′-end of 7SLrem map to an unpaired loop in the full-length 7SL RNA, implying that an unidentified single-stranded endonuclease is responsible for its processing.

The *cis*-acting determinants of 7SL packaging by HIV-1 have been identified *via* exogenously expressed 7SL RNA mutants (Keene and Telesnitsky, 2012). Both Alu (Alu114 nt) and S domains (S114 nt) of 7SL RNA were able to mediate packaging independently (Figure 2B). Endogenous 7SL RNA and Alu domain were packaged through a competitive mechanism while the S domain was packaged efficiently *via* a separated additive mechanism. Interestingly, S114 resembles the 7SLrem fragment, while Alu114 corresponds to the murine 7SL derivative B1 (Onafuwa-Nuga et al., 2005).

The SRP proteins are also involved in the virus-host response. SRP14 has been identified in a proteomic analysis as one of the top 50 proteins interacting with Gag (Engeland et al., 2014). SRP14 levels are up-regulated after expression of the HIV-1 Tat protein, a *trans*-activator involved in binding to the viral long terminal repeat (LTR) (Jarboui et al., 2012). Further evidence of SRP14 involvement in virus-host interactions comes from a porcine orthologue in which knockdown of SRP14 by siRNAs inhibited the replication of the small RNA from Porcine reproductive and respiratory syndrome virus (PRRSV) (Zhao et al., 2017).

SRP9 and SRP54 interact with the HIV-1 Rev, a viral protein that plays a key role in the late phase of virus replication (Naji et al., 2012). Moreover, the Srp9 was identified among 10 genes which expression was reduced in astrocytes upon HIV-1 infection (Su et al., 2003).

Overexpression of SRP19 was associated with the depletion of the antiviral host protein A3G from processing bodies (P bodies), through the impair of 7SL RNA incorporation into virions, but did not affect its HIV-1 virion incorporation and A3F localization (Izumi et al., 2013). This data suggests that the association of A3G with P bodies may be dependent on the presence of 7SL RNA.

In HIV-1 infected cells, SRP68 and SRP72 have been found in cytosolic RNP complexes. These HIV-1 RNPs contain the genomic viral RNA, four viral proteins (Gag, Pol, Env, Nef), the double strand RNA-binding protein Staufen1 and other proteins involved in splicing, metabolism and cell traffic (Milev et al., 2012). During viral infection, HIV-1 protease cleaves SRP72 at amino acid residues A519-L520, within the cleavage site DVEA↓LENS (Impens et al., 2012). While the role of the proteolytic processing of SRP72 is not known, the finding reinforces the idea that modulation of protein translation during infection is probably achieved by proteolytic processing of additional host factors.

### 7SL RNA: Dicer, miRNAs and RBCs

Several ncRNAs are spliced and/or processed into smaller products. 7SL RNA has been reported to be processed into multiples fragments with different regulatory functions. Dicer, a double strand endonuclease involved in miRNAs biogenesis, can process a minor portion of the cellular 7SL RNA pool into fragments of different lengths ranging from 20 to 200 nucleotides (Figure 2C) (Ren et al., 2012). Part of the Dicer-processed 7SL RNA fragments function as dominant-negative regulators of the full-length 7SL RNA, interfering with the formation of SRP complex and inhibiting ER-mediated protein secretion (Ren et al., 2013). A second biological function for Dicer-processed 7SL RNA fragments involves inhibition of miRNA-581 ability to stimulate Hepatitis B virus (HBV) surface antigen (HBsAg) expression (Wang et al., 2014). Interestingly, miRNA-581 targets Dicer expression through translation inhibition, suggesting the existence of a feedback loop between the intracellular levels of 7SL RNA, Dicer and its target miRNAs. The precise mechanism of processed 7SL RNA in inhibiting HBsAg expression remains unknown but it is likely to be mediated by endoplasmic reticulum degradation-enhancing alpha-mannosidase-like protein 1 (EDEM1), a downstream target of miRNA-581.

Recently, 7SL RNA was identified as an abundant component of RBCs of human, mouse and *Xenopus* (Talhouarne and Gall, 2018). The enrichment for 7SL RNA in RBCs is probably due to selective retention during RBC maturation considering that mammalian RBCs lack nuclei and hence do not transcribe RNA. In mammalian RBCs, 7SL RNA is associated with a number of cytoskeletal and membrane-binding proteins, such as spectrin α (SPTA1) and protein 4.1 (Figure 2D). RBCs can contain a short noncoding RNA of 68 nt called sRN7SL, derived from the S domain of 7SL RNA. Production and accumulation of sRN7SL in RBCs is possibly mediated by an unidentified endonuclease. Interestingly, the sequence of sRN7SL overlaps with 7SLrem found in HIV-1 VLPs, suggesting that the S domain of 7SL RNA may carry multiple functions that could be regulated by post-transcriptional processing. A future challenge would be to characterize and understand the roles of other 7SL RNA post-transcriptional modifications. Focusing on how RNA processing contributes to SRP RNA localization and dynamics at the subcellular level would expand its increasing number of cellular functions.

### Alu Domain: Modulator of Translation and Stress Response

The SRP Alu domain encompasses the 5′- and 3′- end of the 7SL RNA forming the Alu motif and the heterodimer SRP9/14. In addition to its role in translation control, as it arrests translation elongation upon SRP binding to translating ribosome, this domain is crucial for transcription, maturation, localization and transport of 7SL RNA (Siegel and Walter, 1988; Lakkaraju et al., 2008).

**FIGURE 3:**
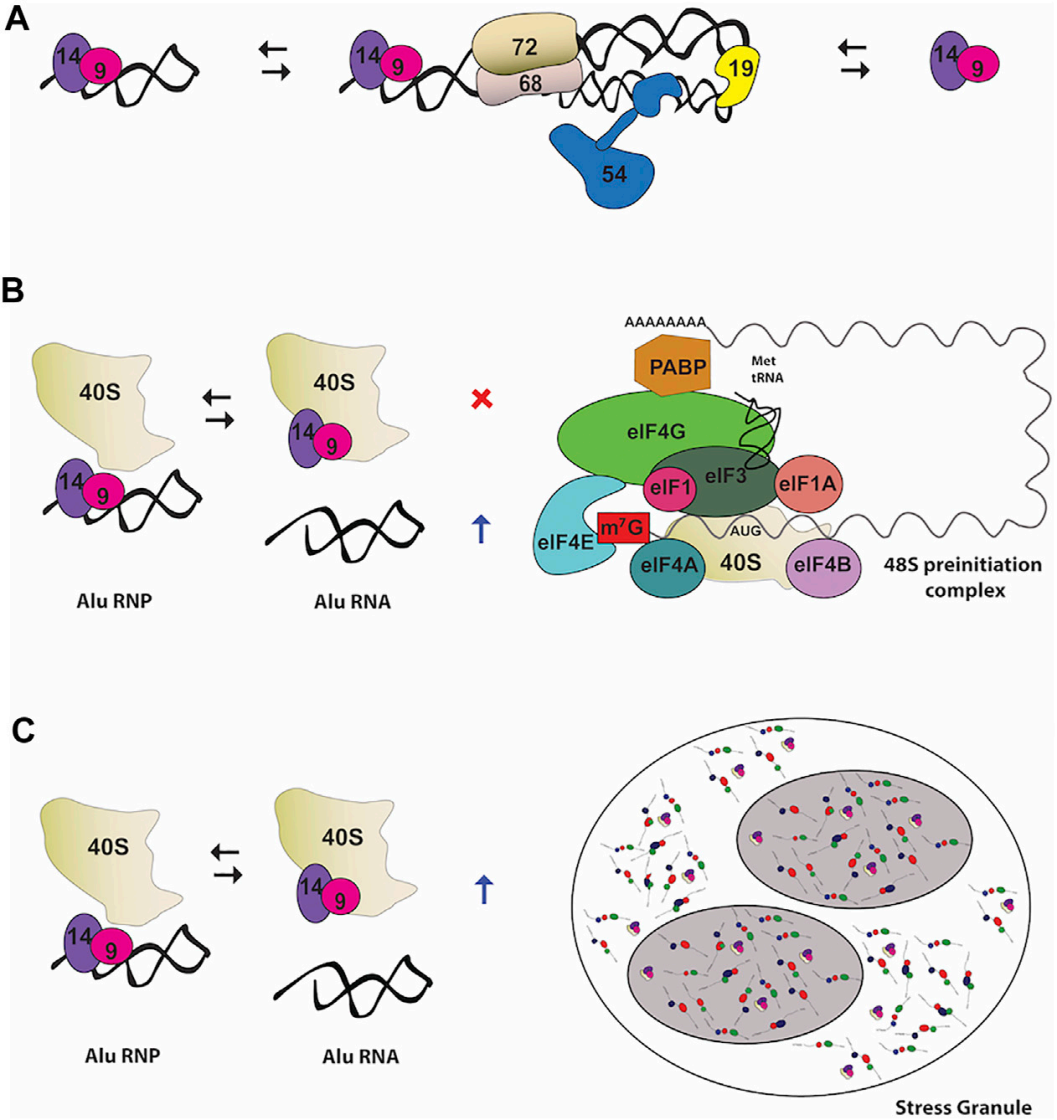
Noncanonical functions of the heterodimer SRP9/14 **(A)** In primate cells, SRP9/14 exists as part of the SRP complex, as well as free protein and in RNP complexes with 7SL RNA-derivatives RNA, such as Alu RNA **(B)** Role of Alu RNP in regulating translation. Alu RNPs interfere with the formation of 48 S preinitiation complex by SRP9/14 binding to the ribosomal subunit 40 S. On the other hand, free Alu RNA stimulates translation by sequestering SRP9/14 **(C)** Role of Alu RNP in stress granules (SGs) assembly. SRP9/14 localizes to SGs through binding with the ribosomal subunit 40 S. Binding of SRP9/14 to 40 S and Alu RNA is mutually exclusive.

SRP9 and SRP14 form a stable heterodimer that can exist free in the cell or bound to the 7SL RNA with high specificity. The heterodimer can bind to a variety of small cytoplasmic ncRNAs structurally and phylogenetically related to the Alu motif of SRP RNA forming an assortment of Alu RNPs (Figure 3A) (Strub and Walter, 1990; Bovia et al., 1995; Chang et al., 1996; Bovia et al., 1997). Alu RNPs inhibit polysome formation through targeting SRP9/14 to a functional site in the 40S ribosomal subunits, interfering with 48S complex formation and translation initiation (Figure 3B) (Chu et al., 1998; Hasler and Strub, 2006). They inhibit translation of cellular mRNAs, as well as, viral IRES-mRNAs, modulating the translational output in response to stress and viral infection (Ivanova et al., 2015).

One of the many Alu-like RNAs that SRP9/14 binds with high affinity is the brain cytoplasmic 200 RNA (BC200 RNA) (Bovia et al., 1997; Kremerskothen et al., 1998). BC200 is specifically expressed in neurons localizing at somatodendritic region and in a number of tumors such as lung and breast carcinomas (Cheng et al., 1997; Kuryshev et al., 2001). BC200 exists as an 11.4 S RNP complex bound to SRP9/14 that acts as a regulator of decentralized translation in neuronal dendrites (Kremerskothen et al., 1998). Not only the inhibitory activity of BC200 RNAs in translation is enhanced by SRP9/14 but these SRP components are involved in stabilization and nuclear export of BC200.

SRP9/14 can be found in stress granules (SG) in response to stress (Figure 3C) (Berger et al., 2014)*.* SG are very dynamic cytoplasmic structures composed of 40 S ribosomal subunits, translation initiation factors, mRNAs, RBPs and other signaling molecules. The expression level of the SRP9/14 determines the size and the number of SG-positive cells due to their involvement in formation and disassembly of SG upon binding to the 40S ribosomal subunit (Berger et al., 2014). The mutually exclusive binding of SRP9/14 onto the Alu RNA and 40S regulates stress response by controlling SG levels. During stress recovery the concentration of cytoplasmic Alu RNA increases and promotes disassembly of SG by sequestering SRP9/14 (Berger et al., 2014).

Future studies should address the functional effect of SRP9/14 during cellular stress responses to human physiology and diseases.

Interestingly, SG and neuronal granules share many components, with BC200 involved in dendritic transport of mRNA granules in neurons, suggesting that SRP9/14 may be involved in a more generic conserved mechanism of mRNP compartmentalization (Zalfa et al., 2005; Cao et al., 2006).

Large-scale identification and characterization of other SRP9/14-RNP complexes would be beneficial in unveiling more SRP-independent functions associated with this heterodimer. The analysis of the proteome and transcriptome associated with SRP9/14 could shed light on the mechanism underlying the recruitment of these components.

### The Heterodimer SRP68/72: Transcription Regulation and Histone Networks

SRP68 and SRP72 form a stable heterodimer that assembles into the pre-SRP complex in the nucleus and is necessary for export to the cytoplasm (Grosshans et al., 2001). SRP68/72 was reported to bind chromatin and regulate transcription in an SRP independent manner (Li et al., 2012). Upon interaction with the tail of histone H4 in a methylation-sensitive manner, SRP68/72 regulates several gene expression indicating its role and interaction with other cellular components outside the protein synthesis cycle.

**FIGURE 4:**
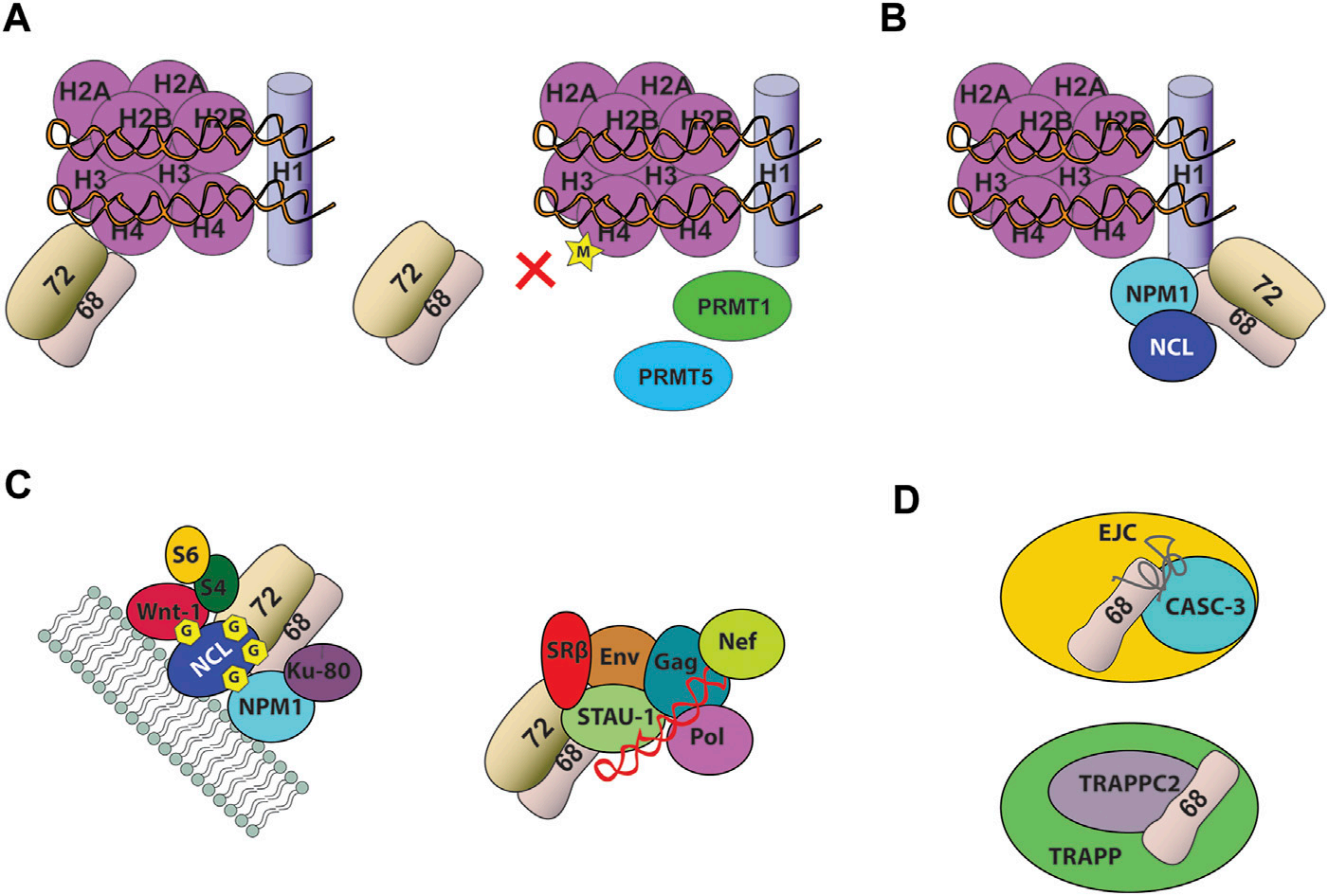
Noncanonical function of the heterodimer SRP68/72 **(A)** SRP68/72 binds the tail of H4. Dimethylation of H4 by PRMT1 and PRMT5 causes SRP68/72 chromatin dissociation and shuttle in the cytoplasm **(B)** SRP68/72 binds C-terminal domain of H1 linker histone **(C)** SRP68/72 heterodimer was identified in different RNPs. It was found associated with cell-surface nucleolin (NCL) in a highly stable 500-kDa protein complex including several proteins (left) and in Staufen1-RNP complex (right), including four viral proteins (Gag, Env, Pol, Nef), the viral RNA (shown in red) and more than 200 other proteins **(D)** SRP68 was found associated to CASC-3 and TRAPPC2, respectively in EJC and TRAPP particles. Depicture of interactions in complexes are schematic only.

SRP68/72 can only bind to non-methylated or mono-methylated H4. The post-translational marks H4R3me2a and H4R3me2s by the protein arginine methyltransferases (PRMT1 and PRMT5) have an inhibitory effect on SRP68/72-H4 tail binding (Figure 4A). Consistently, exogenous expression of PRMT1 and PRMT5 results in the dissociation of SRP68/72 from the chromatin and its export to the cytoplasm.

SRP68 and SRP72 proteins can transcriptionally activate a promoter-driver luciferase reporter, indicating for the first time a transcriptional regulation function for the SRP (Li et al., 2012). This activity was mapped into their protein-binding domain, corresponding to the C-terminus of SRP68 and the N-terminus of SRP72. ChIP-seq experiments reported that SRP68 binds genes involved in cell adhesion, cytoskeletal organization, DNA catabolism and apoptosis. Knockdown of SRP68 differentially affected the expression of its associated target genes, suggesting a context-dependent transcriptional function for SRP68. Future directions should focus on the transcriptional activity of SRP72, including the identification of genes that bind to SRP72, as well as the genes affected by SRP72 levels.

Two proteomic studies focused on characterizing the interaction network of H1 linker histones identified SRP68 and SRP72 as possible binding partners (Figure 4B) (Kalashnikova et al., 2013; Zhang et al., 2016). The H1 linker histones are a family of chromatin-associated DNA-binding proteins that function by stabilizing nucleosome structures and condensed states of chromatin (Woodcock et al., 2006). The majority of H1-binding proteins are components of the nucleolus, suggesting that SRP68/72 and H1 interaction happens in this cellular compartment. SRP68/72 can bind to at least three different variants of H1 (H1.1, H1.2, and H1. X) throughout the cell cycle, which may contribute to dynamically regulate linker histones. The SRP68-H1 interaction is mediated by the C-terminal domain of H1, an intrinsically disordered domain, however no structural information regarding SRP and histone interactions is available (Lu et al., 2009). Currently the mechanism and the functional nature of the interaction between SRP68/72 and histones, such as H1 and H4, have not yet been fully elucidated and further links between SRP68/72 and histones need to be unveiled.

### The Heterodimer SRP68/72: Multiple Noncanonical Binding Partners

SRP68/72 heterodimer associates with cell-surface nucleolin in a stable 500-kDa protein complex that includes proteins involved in cancer progression, such as nucleophosmin (NPM1), Wnt-1, the antigen Ku80 and C1q-R, as well as the ribosomal proteins S4 and S6 (Figure 4C) (Krust et al., 2011; Destouches et al., 2012). Nucleolin is a multifunctional DNA-, RNA- and protein-binding protein that shuttles between different cellular compartments (Ginisty et al., 1999). At the cell surface, nucleolin serves as anchoring site for several ligands involved in cell proliferation, apoptosis, angiogenesis and as a low affinity receptor for HIV-1. Surface expressed nucleolin is glycosylated and constantly induced in tumor cells, as well as, activated endothelial cells and has been validated as a novel target in anticancer therapy (Srivastava and Pollard, 1999). The role of the association of SRP68/72 with cell-surface nucleolin is still unknown but these findings may indicate that SRP68/72 can coordinate the active translocation of nucleolin toward the plasma membrane or that nucleolin can regulate the nucleocytoplasmic shuttle of SRP68/72. Noteworthy, nucleolin and nucleophosmin are two histone chaperones, associated with both core and linker histones. Considering the link between SRP68/72 and histones, it is possible to envisage that SRP68/72 interaction with histones could be regulated by a nuclear binding partner, such as nucleolin and nucleophosmin. In addition, Ku80 and nucleolin are important autoantigens in patients with systemic lupus erythematosus and other systematic autoimmune disorders, suggesting a potential implication of SRP68/72 associated with the 500-kDa complex in autoimmune diseases. Finally, in HIV-1 infected cells SRP68/72 and the SRP receptor SRβ have been found associated with nucleolin in Staufen1-RNP (Figure 4C), indicating that nucleolin and SRP68/72 interaction has a wider role in multiple RNPs (Milev et al., 2012). Nucleolin seems to be a key mediator in modulating SRP68/72 noncanonical functions and could be a potential candidate involved in shuttling this SRP heterodimer inside the cells.

Recently, SRP68 has been identified as a component of CASC3-containing exon junction RNP complex (EJC) through a weak RNA-interaction (Mabin et al., 2018) and as a binding partner of the TRAnsport Protein Particle complex subunit 2 (TRAPPC2), a trafficking complex localized in perinuclear granular structures (Figure 4D) (Zappa et al., 2019).

Although the roles of SRP68 in these multimolecular complexes remain to be defined, SRP68 could be involved in the recruitment of CASC3 and TRAPPC2 by a “piggyback” mechanism involving interaction with RNA, RBPs and RNPs components of EJC and SGs, respectively.

Identification of more noncanonical binding partners that can bind SRP68/72 and the functional and structural characterization of these interactions would clarify the different biological roles of SRP68/72 and its interplay with other RNPs. However, the instability and insolubility of SRP68/72 causes the purification of these proteins to remain challenging, contributing to the lack of crystallographic structural information of full-length proteins, to date. Moreover, the transient and highly dynamic nature of many RNPs makes structure determination a more arduous task.

### SRP72 and Apoptosis

**FIGURE 5:**
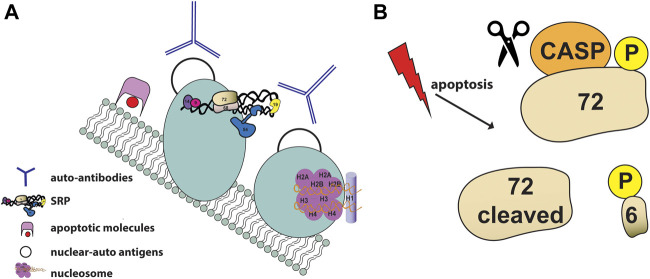
SRP and apoptosis **(A)** Schematic illustration of SRP autoantigens as constituents of membrane-bound blebs (shown as light blue circles) on the surface of apoptotic cells. Apoptotic blebs display many nuclear auto-antigens (shown as black empty circles) such as nucleosomes (shown in purple) and accumulate apoptotic molecules (shown in pink and red). Auto-reactive antibodies (shown as blue Y) generated against nuclear antigens are showns around the blebs **(B)** SRP72 is phosphorylated and cleaved during apoptosis. The proteolytic cleavage of SRP72 by caspases produces a 6 kDaC-terminal phosphorylated peptide of unknown fate.

Post-translational modifications of proteins have been proposed by LeFeber to be essential to the development of autoantibodies and autoimmune diseases, leading to the hypothesis that modifications of autoantigens during apoptosis can cause the development of autoantibodies upon bypassing normal mechanism of tolerance (Lefeber et al., 1984; Utz and Anderson, 1998; Utz et al., 1998). For instance, Lupus autoantigens such as Ro, La and the U1-70 kDa proteins have been shown to localize to cell surface apoptotic blebs following UV-irradiation (Casciola-Rosen et al., 1994). SRP autoantigens have been identified as constituents of membrane-bound blebs on the surface of apoptotic cells, the ideal location for presentation to the immune system (Figure 5A). SRP components were found as constituents of discrete small cell surface apoptotic blebs (≈1.4 μm in diameter), including the ribonucleoprotein autoantigen Ro, calreticulin and the ER luminal marker BiP, in epidermal keratinocytes after UVB irradiation (Casciola-Rosen and Rosen, 1997).

The first direct evidence of the involvement of the SRP in apoptosis and the description of post-translational modifications of the SRP autoantigen complex was reported in 1998 by Utz and colleagues (Utz et al., 1998). Using sera from patients with different autoimmune diseases, SRP72 was the only SRP component identified to be phosphorylated and cleaved during apoptosis (Figure 5B) (Utz et al., 1998). Despite the fact that SRP72 proteolytic cleavage can be prevented with caspase inhibitors, the caspase responsible for cleaving SRP72 remains unknown. SRP72 has a group III caspases putative cleavage site (_614_SELD/A_618_) followed by highly conserved serine residues that produces a 6 kDaC-terminal phosphorylated peptide. The fate of the phosphorylated peptide remains unknown, however selective phosphorylation of SRP72 suggests that a serine kinase and/or a phosphatase regulates SRP72 levels.

Caspase-mediated cleavage of certain proteins during apoptosis has been shown to contribute directly to the apoptotic phenotype (Rudel and Bokoch, 1997). Currently it is still unknown whether phosphorylation or caspase-mediated cleavage of SRP72 contributes to the production of autoantibodies reactive with components of the SRP complex. The cleavage of SRP72 may play a critical role when apoptosis is induced by envelop virus, since many viral proteins pass through the ER, suggesting that SRP72 cleavage may protect neighboring cells by preventing viral replication and shedding.

In addition, a correlation between SRP and TNF related apoptosis inducing ligand (TRAIL)-death- receptor pathway reinforces the involvement of SRP in apoptosis and reveals a mechanistic relationship between protein trafficking and cell death signaling (Ren et al., 2004). TRAIL signaling plays a main role in tumor immune-surveillance by inducing selectively apoptosis in cancer cells and sensitivity of cancer cells to TRAIL correlates with the surface level of death receptors DR4 and DR5 (Zhang et al., 1999). A pro-apoptotic role of SRP is necessary for DR4-mediated apoptosis and sensitivity in cancer cells. Silencing SRP54 or SRP72 by RNAi inhibits DR4-mediated apoptosis by decreasing DR4 cell surface localization and blocking the TRAIL cascade at the apical point, before procaspase-8 processing. Interestingly, SRP silencing had no effect in DR5 cell surface localization, indicating a specificity in regulating DR4-pathway instead of DR5 and other TNF receptors.

Future studies are needed to unravel the mechanistic details of SRP involvement in apoptosis. A starting point would be the characterization of the cellular pathway that leads to SRP72 phosphorylation and cleavage and the relation between SRP and extrinsic signaling through death receptors.

## SRP and Diseases

With multiple functionalities within cells, deregulation of SRP expression results in diseases and malignancy, suggesting that controlling the expression levels of SRP components is key to normal cellular function (Table 2). SRP autoantibodies have been found in the serum of patients with a wide range of myopathies. SRP components have been involved in noncanonical pathways, cancer and other diseases, suggesting a new perspective in using SRP components as biomarkers. Multiple proteomic and genomic studies report alteration in gene expression of SRP components, however only 7SL RNA has been shown to have a direct role in cancer progression.

**TABLE 2:** Diseases and biomarkers associated with SRP subunits.

SRP component	Autoimmune diseases	Expression in cancer	Genetic diseases	Targets and biomarker	References
7SL RNA	Idiopathic inflammatory myopathies (IIM), such as dermatomyositis, polymyositis and necrotizing autoimmune myositis	↑ liver, lung, breast and stomach cancer		oncogene	White (2004); Abdelmohsen et al. (2014)
SRP9		↑ metastatic human prostate cancer, hepatocellular and colorectal adenocarcinoma		gastric cancer	Liu et al. (2007); Rho et al. (2008); Ye et al. (2020)
				RFS in multiple sclerosis	
SRP14		↑ metastatic human prostate cancer and KRAS-positive cells		gastric cancer	Kundu et al. (2012)
SRP19		↓ bladder cancer cells		colon cancer that harbour APC loss	Tsai et al. (2014); Rosenbluh et al. (2016)
SRP54		↑ metastatic human prostate cancer cells	Congenital neutropenia with Shwachman-Diamond-like phenotype	kidney transplant outcome	Srivastava et al., (2015); Bellanne-Chantelot et al. (2018)
SRP68		↑ breast and bladder cancer cells	Congenital neutropenia	RF radiation	Zhang et al. (2013)
SRP72		↑ breast, thyroid and prostate cancer cells	Aplastic anemia, myelodysplasia	radiosensitivity	Tiwana et al. (2015); Chai et al. (2016); Vatakuti et al. (2016)
				thyroid oncogene	
				drug-induced hepatotoxicity	

To date, mutations associated with genetic diseases have been reported for three components of the SRP. SRP72, SRP54 and SRP68 display mutations associated with bone marrow failure, opening a new window into the pathophysiology of SRP and genetic diseases.

### SRP Autoantibodies and Autoimmune Diseases

SRP has been implicated in the progression of different idiopathic inflammatory myopathies (IIM), such as dermatomyositis, polymyositis and necrotizing autoimmune myositis. IIM are chronic autoimmune muscle diseases characterized by muscle weakness, inflammation in skeletal muscle tissues and by the presence of autoantibodies against specific cellular components, particularly RNP complexes (Findlay et al., 2015; Lundberg et al., 2016).

Autoantibodies targeting SRP are subjects of considerable clinical interest and were first described in 1986 (Reeves et al., 1986). Human antibodies against SRP immunoprecipitated the 7SL RNA and all the SRP proteins. Among all components of SRP, SRP54 is considered the main antigenic target of anti-SRP antibodies (Targoff et al., 1990) with antibodies against SRP54 found in patients with severe myopathies and Lupus-type autoimmune diseases (Miller et al., 2002; Hengstman et al., 2006). Specific SRP54 antibody inhibitory effect is on translocation upon interference with signal sequence binding and SRP receptor interaction, most likely by steric hindrance (Romisch et al., 2006).

The mechanism of anti-SRP associated myopathy is mediated in a complement-dependent antibody as suggested by SRP co-localization on the cell surface with C3c, the activated product of the central component of the complement system C3 (Rojana-Udomsart et al., 2013). At the cell surface, SRP can bind to anti-SRP antibodies forming antigen-antibody complexes that activate the membrane attack complex C5b-9. Surface redistribution of intracellular ribonuclear antigens, such as Ro and La, has been shown to occur in skin cells undergoing apoptosis, however the mechanism responsible to the presence of SRP in cell surface remains unknown (Casciola-Rosen et al., 1994; Herrera-Esparza et al., 2006).

Whether or not SRP is associated with a specific form of immune-mediate myopathy is still under debate. Anti-SRP autoantibodies were specific for severe polymyositis, necrotizing myopathy and sometimes detected in patients with other autoimmune syndromes such as rheumatoid arthritis, inflammatory bowel disease and systematic sclerosis (Miller et al., 2002; Kao et al., 2004).

The presence of anti-SRP autoantibodies is associated with a unique spectrum of clinical phenotype called “the anti-SRP syndrome” with significance in diagnosis, management and prognosis of the disease (Targoff, 2000). Based on clinical observation, the presence of anti-SRP antibodies correlates with a broad acute onset of severe features, such as progressive proximal weakness, dysphagia and resistance to standard treatments. Anti-SRP positive patients have a rarity or absence of foci of mononuclear inflammatory cells (Miller et al., 2002).

Anti-SRP autoantibodies were found in patients with necrotising myopathy with a rapid progressive course and severe disability (Hengstman et al., 2006). In inflammatory myopathy, anti-SRP antibodies are associated with different clinical courses and histological presentation, including severe limb weakness, muscle atrophy and poor neurological outcomes (Suzuki et al., 2015). Anti-SRP19 antibodies were present in skeletal muscle tissues of patients with autoimmune necrotizing myopathy (Wang et al., 2016). SRP19 antibodies expression was found mainly in necrotic myofibers, the stroma cells and the surrounding small blood vessels. Interestingly, the presence of anti-SRP19 antibodies in non-necrotic myofibers suggests a possible role of SRP19 in the early stages of the disease.

The most common cause of death in IIM patients are cardiovascular manifestations and cancer (Danko et al., 2004; Garcia-De La Torre, 2015). An association between anti-SRP and cardiac disease has been proposed with anti-SRP antibodies found in cases of cardiac involvement in the form of arrhythmia and cardiomyopathy (Hirakata et al., 1992). A comparative study showed that anti-SRP positive patients with polymyositis had cardiac symptoms that included edema, dyspnea on exertion, and chest pain, but the cardiac involvement was considered statistically nonsignificant (Hengstman et al., 2006). More recently, in juvenile IIM, the presence of anti-SRP was associated with more frequent abnormal ECG and by echocardiography (Rider et al., 2013). Three single nucleotide polymorphisms (SNPs) located in the SRP54 antisense gene were found to be associated with cardiovascular diseases in patients with systemic lupus erythematosus but not with rheumatoid arthritis (Leonard et al., 2018), however, further studies are necessary to clarify the risk of cardiac involvement in SRP-myopathy.

Cancer can occur in 20% of patients with IIM, especially during dermatomyositis (Zahr and Baer, 2011). Cancers were detected in 5–17% of patients with anti-SRP myopathy, however no significant difference was found in clinical features between SRP-cancer and SRP-cancer free patients (Allenbach et al., 2016; Kadoya et al., 2017).

### SRP Components as Biomarkers in Cancer

7SL RNA is up-regulated in several cancer cells and its silencing is correlated with reduction in cell proliferation (White, 2004). Analysis of 80 tumor specimens representing 19 types of cancer revealed that 7SL RNA is abnormally abundant in every tumor analyzed, including liver, lung, breast and stomach cancer (W. Chen et al., 1997). The proliferative effect of the 7SL RNA is due by its interaction with the 3′untranslated region (3′UTR) of TP53 mRNA, which encodes for the tumor suppressor p53 (Abdelmohsen et al., 2014)*.* This interaction reduces p53 translation preventing the recruitment of the RNA-binding protein Human antigen R (HuR) to TP53 mRNA. Silencing of 7SL RNA increases p53 translation through recruitment of HuR, suggesting a mechanism in which 7SL and HuR compete for binding TP53 3′UTR. Transcription of 7SL RNA is repressed by the tumor suppressor FOXP3, contributing to tumor growth inhibition (Yang et al., 2016)*.* ChIP analysis showed that FOXP3 directly binds to the 7SL RNA promoter regions, through Forkead/HNF-3 domain DNA binding sites. Repression of 7SL RNA by FOXP3 promotes the expression of the downstream target p53 protein, indicating a feedback loop between FOXP3, p53 and 7SL.

In breast cancer, the 7SL RNA has been found in extracellular vesicle (EV)-mediated cell transfer (Nabet et al., 2017). EVs are mobile membrane-limited vesicles, which contains proteins, RNA and lipids derived from the cytoplasm of their donor cell and can transfer their contents intercellular, providing a mechanism of cell-cell communication (Gyorgy et al., 2011). Since 7SL RNA and its correlated RN7SL1 pseudo-genes were found in stromal cell-derived EVs, it could be implied a potential role for 7SL RNA in acting as a damage-associated molecular pattern (DAMP) in the crosstalk between stromal cells and breast cancer cells. Transfer of 7SL RNA by activated stromal EVs to adjacent triple-negative breast cancer cells stimulates anti-viral signaling, resulting in enhanced tumor growth and a malignant phenotype (Nabet et al., 2017). Specifically, stromal 7SL RNA stimulates the viral RNA pattern recognition receptor (PRR) RIG-I, resulting in STAT1 activation and interferon-stimulated genes (ISG) induction. Specific bind of 7SL RNA to the C-terminal domain (CTD) of RIG-I is mediated by the presence of the common RNA viral structural motif 5′-triphosphate (5′ppp) at its end.

Multiple studies analysing differential gene and protein expression profile have identified SRP proteins associated with cancer or other diseases. SRP9/14 have been involved in cancer development. SRP9 mRNA was found up-regulated in hepatocellular carcinoma *via* gene array studies (Liu et al., 2007) while a proteomic analysis has shown it was overexpressed in patients with colorectal adenocarcinoma (Rho et al., 2008), suggesting SRP9 as a candidate biomarker for colon cancers. Two recurrent nonsynonymous RNA editing events that recode the amino acid sequence of SRP9 were observed in colorectal cancer and in primary breast cancer (Shah et al., 2009; Lee et al., 2017). Additionally, SRP9 protein was found overexpressed in small lymphocytic lymphoma (SLL) (Rolland et al., 2011). SRP14 expression was enhanced in cell lines positive for the KRAS-variant, an oncogene frequently mutated in various types of cancer (Kundu et al., 2012). The heterodimer SRP9/14 levels increased in the serum of the H22 hepatoma-bearing mice and its expression reduced by the antitumoral *Scutellaria barbata* polisaccharides (Li et al., 2019).

SRP19 has been associated with colon and gastric cancer. Srp19 gene is in close proximity to the tumor suppressor adenomatous polyposis coli (APC) gene, which is often deleted in ß-catenin-active cancers (Joslyn et al., 1991). SRP19 mRNA and protein levels were lowered in cell lines harboring APC deletion and shRNA targeting SRP19 inhibited proliferation of cells, confirming Srp19 as a CYCLOPOS gene in colon cancer with APC loss (Rosenbluh et al., 2016). Srp19 gene has been associated with gastric cancer, with a significantly lower copy number compared to healthy patients and may serve as a potential novel biomarker to identify high-risk individuals (Tsai et al., 2014). Regulation of the expression of SRP genes and proteins in liver and colon cancer remains to be elucidated; however, alterations in the expression levels of SRP9/14 and SRP19 indicate a key role for SRP in gastro-related cancer.

Srp68 and Srp19 genes were among 516 genes differentially expressed in bladder cancer and reported to be up-regulated and down-regulated respectively (Zekri et al., 2015). Srp68 and Srp72 genes were identified as potential nuclear LIM-only protein 4 (LMO4)—target genes in human breast cancer cells (N. Wang et al., 2007).

Few evidences of association between SRP and prostate cancer were also reported. SRP9, SRP14 and SRP54 were overexpressed in metastatic prostate cancer cells treated with curcumin, an ER stress-mediated pro-apoptotic and antitumoral molecule extracted from the turmeric rhizome (Rivera et al., 2017). In prostate cancer, Srp72 gene was identified as a downstream target of the cancer biomarker TWIST, associated with cancer invasion and metastasis (Lyu et al., 2017). Srp72 gene was a recurrent mutated cancer-driving gene in thyroid cancer (Chai et al., 2016). Srp72 is overexpressed and hypomethylated which suggests it may have oncogenic function in thyroid cancer by altering the methylation status. A deletion in Srp72 gene (4q22.3) was also implicated in copy number variation in thyroid cancer, suggesting that this gene may contribute to the formation and progression of thyroid cancer in various ways (Chai et al., 2016).

### SRP Role in Other Diseases and Noncanonical Pathways

SRP genes and proteins were found specifically involved in a multitude of diseases and pathways. Interestingly, a comparative proteomic study of inflammatory vs. angiogenic activated endothelial cells identified all the SRP components to be overexpressed upon interleukin 1 beta (IL1B) and vascular endothelial growth factor (VEGF) treatment (Mohr et al., 2017) suggesting a role of SRP in angiogenesis and inflammatory pathways. Srp9, Srp14 and Srp54 genes were up-regulated in patients with thyroid-associated ophthalmopathy (TAO), suggesting that SRP might play a role in the development of TAO (Zhao et al., 2015).

A transcriptomic approach using human precision-cut liver slices, identified Srp72 among with different ER stress genes as a marker phenotype of drug-induced hepatotoxicity, confirming the involvement of ER stress in cholestasis and liver toxicity (Chen et al., 2014; Vatakuti et al., 2016). Recently, SRP72 was identified as a radiosensitizer and has been proposed as a modulator of radiosensitivity in mammalian cells (Tiwana et al., 2015). SRP72 depletion was associated with a decreased radiosurvival after irradiation and correlated with a delayed G2/M cell arrest, elevated level of apoptosis and unfolded protein response (Prevo et al., 2017). Due to healthy cells also being affected by SRP72 depletion, a drug inhibition of SRP72 is unlikely to be a useful strategy for radiotherapy treatment, but SRP72 levels can be a biomarker for radiosensitivity. Interestingly, an independent study reported that SRP68 expression decreased significantly, suggesting it as a biomarker, in human lens epithelial cells exposed to radiofrequency radiation (Zhang et al., 2013).

Srp19 gene was found up-regulated in patients with Kashin-Beck disease (KBD) and KBD accompanied with dental fluorosis (DF) (Zhang et al., 2018). In another study, Srp19 gene was identified as a nitric oxide-sensitive gene and found down-regulated in human monocytic cells after exposure of nitric oxide (Turpaev et al., 2010). SRP54 protein was identify among the top-ranking five non-invasive biomarkers that can accurately predict kidney transplant outcome and its down-regulation presenting a detrimental profile common for both rejection and injuries (Srivastava et al., 2015).

A more recent function of SRP in brain diseases has emerged recently. Srp54 gene expression was strongly down-regulated in response to anti-inflammatory and anti-depressant drugs (Ulrich-Merzenich et al., 2012) and a single nucleotide polymorphism (SNP) in Srp54 has been associated with bipolar affective disorder (Baum et al., 2008). Moreover, in neurons, SRP54 was reported as a novel dopaminergic transcription factor which significantly enhances the promoter activity of the dopamine transporter (DAT) gene SLC6A3 (Zhao et al., 2018).

Srp9 was identified as a febrile seizure (FS) susceptibility gene and its overexpression found to increase FS susceptibility through ER-dependent synthesis and trafficking of membrane proteins, such as glutamate receptors (Hessel et al., 2014). Furthermore, Srp9 gene was reported to contribute to neuroticism, a high-order personality trait (Ohi et al., 2017). It was found to be intensively enriched in brain and associated with the glutamate receptor activity network. Recently, Srp9 gene was found to be part of a five-gene signature proposed as a novel marker tool to predict the relapse-free survival (RFS) in patients with multiple sclerosis (Ye et al., 2020).

The role of SRP19 and SRP54 in neuronal diseases, such as bipolar affective disorder and neuroticism, need to be eluciated and warrant future investigation. However, it can be postulated that the glutamate network might be a key target of SRP in the brain.

Finally, a transcriptome profile study revealed that transcripts related to translation, including the cytoplasmic non-coding RNA 7SL, were enriched in the axons of motoneurons, indicating for the presence of the protein secretion machinery in axons (Briese et al., 2016).

### SRP Genetic Diseases and Trans-splicing Events

Autosomal-dominant mutations in SRP72 were identified in the pathophysiology of aplastic anemia (AA) and myelodysplasia (MDS), two forms of bone marrow failure that are associated with an increased risk of hematologic malignancy (Kirwan et al., 2012; Galaverna et al., 2018). Two different heterozygous SRP72 mutations were found in two families: a missense mutation in exon 6 (R207H), near the middle of the sixth TPR domain, and a deletion in its RNA-binding domain (T355deletion). The mutated SRP72 protein localizes incorrectly within the cell, with impaired ability to assemble onto the SRP complex. These finding support a link between a defect in protein translocation and AA/MDS.

The molecular role of SRP in the hematopoietic system and its involvement in aberrant haematopoiesis is currently unknown. Recently, a Srp72 deficient mouse model was developed to characterize the impact of heterozygous loss of Srp72 on murine haematopoiesis (D'altri et al., 2019). In contrast to the severe phenotype observed in two families, heterozygous loss of Srp72 in mice was associated only to a mild haematological phenotype. Despite this, modulation of SRP72 levels influenced the transcriptional down-regulation of key hematopoietic cytokines and receptors.

Autosomal dominant mutations in SRP54 were reported to cause syndromic congenital neutropenia (CN) with a Shwachman-Diamond-like phenotype (SDS), a rare inherited bone marrow failure disorder (Carapito et al., 2017; Bellanne-Chantelot et al., 2018; Goldberg et al., 2020). Four different mutations were identified (T115A, T115deletion, T117deletion, G226E) from SDS patients, all of them affecting highly conserved amino acids within the G-domain of SRP54. The T115A, T115deletion and T117deletion impair SRP54 GTPase activity function, while the G226E affects SR receptor binding, leading to ER stress and autophagy (Juaire et al., 2021).

SRP54 mutations are not restricted to SDS patients and could be involved in the generic mechanism of neutropenia. In fact, mutations in SRP54 are the second most common cause of severe inherited neutropenia in the French Congenital Neutropenia Registry (Oyarbide and Corey, 2018).

At the beginning of 2021, the first *in vivo* model of SRP54 deficiency in zebrafish indicated that SRP54 is critical for the development of multiple tissues and its mutations impair neutrophil differentiation by hampering the unconventional splicing of the transcription factor XBP1 (Schürch et al., 2021).

Recently, SRP68 has been implicated as well in a sporadic case of severe CN (Schmaltz-Panneau et al., 2021). A large intronic homozygous deletion of exon 1 (c.184+2T>C) of SRP68 gene was found responsible to trigger an ER stress resulting in an increased P53-dependet apoptosis of granulocytic precursors and neutrophils.

Alternative splicing involving SRP19 and APC genes has been reported (Kinzler et al., 1991; Horii et al., 1993) with the intergenic *trans*-splicing being controlled in a tissue-specific manner, but the role of the chimera product remains unknown. Interestingly, another trans splicing event involving SRP72 and Calcium/calmodulin-dependent protein kinase II (CaM II) produced a chimera isoform of CaM II kinase called γSRP in human islets of Langerhans (Breen and Ashcroft, 1997). The γSRP isoform retains most of the catalytic properties of CaM II, however its functional role still needs to be identified.

Alternative spliced transcript variants encoding multiple isoforms have been observed for SRP68 gene. Three pseudogenes of Srp68 gene are located within the Smith-Magenis syndrome (SMS) region on chromosome 17 (Park et al., 2002). This region is believed to mediate nonallelic homologous recombination, resulting in both SMS deletions and reciprocal duplications (K.-S. Chen et al., 1997).

### SRP as a Drug Target

Recently, the bacterial SRP was proposed as a potential target for developing a new class of antibiotics (Faoro et al., 2018; Ghosh et al., 2018). However, to date, the human SRP has not been explored directly as a potential target for drug discovery.

A number of inhibitors/drugs such as aflatoxin B1 (AFB1), leptomycin B and ivermectin have been shown to either interact with SRP components or inhibit its nuclear export/import process (Alavian et al., 2004; Wagstaff et al., 2012). The hepatocarcinogen AFB1 has been shown to interact with components of the SRP Alu domain and to interfere with the formation of functional SRP S domain (Singh et al., 2005; Singh et al., 2006). The mechanism of action of AFB1 on SRP is selective to bind SRP9/14 and to inhibit the expression of SRP54 and SRP72.

Currently, the first and only anticancer drug reported to target the SRP is TAS-103, a quinoline derivative that displays antitumor activity in murine and human models (Utsugi et al., 1997; Byl et al., 1999; Ohyama et al., 1999). TAS-103 specifically binds to SRP54 disrupting SRP formation and reduces the expression of SRP14 and SRP19 (Yoshida et al., 2008). Functional studies are needed to investigate the interaction of SRP and TAS-103 and the role of SRP disruption in cancer for drug discovery, indicating a much larger range of drug targeting potential toward the SRP components.

Due to the SRP multifunctionality and its vital role in cellular processes, there is a potential to develop therapies targeting specifically some of the SPR components noncanonical functions. From a structural point of view, the identification and characterization of the binding interface of accessory proteins and SRP components could be exploited with small molecules or RNA analogues to target specific interactions in the cell that could be potentially linked to diseases. Protein-protein interactions are challenging targets, as they can overlap with the SRP-RNA binding sites, however, some of these binding interfaces may turn into prospective targets for drug design if they do not interfere with the canonical function of SRP. For the therapeutical point of view, detailed studies focusing on the individual interaction interface between SRP components and their noncanonical counterparts are needed to drive the drug discovery process.

In particular, the 7SL RNA is an interesting target for the development of new anticancer drugs. The recent identified interaction between the 7SL RNA and TP53 mRNA could be targeted for the treatment of cancers with reduced p53 levels. Promising, the influence of 7SL on p53 expression is likely independent of its function as part of the SRP (Abdelmohsen et al., 2014).

Whether 7SL also influences the expression of other proteins involved in proliferation of cancer cells remains to be investigated. Future research topic should focus in elucidating the function of the 7SL RNA in cancer, investigating its tissue-specific interaction with other RNAs and the possibility of targeting these RNA-RNA interactions. Finally, a murine model system of 7SL RNA would be beneficial to evaluate the efficacy and safety of different treatments for cancer.

Furthermore, targeting the noncanonical role of SRP9/14 in SGs formation and disassembly could be a potential therapeutic strategy for the treatment of many human diseases where SGs are mainly involved, such as pathogenesis of neurodegenerative diseases, viral infection, aging and cancers (Wang et al., 2020). For instance, a promising field to explore would be the identification of small molecules that target SRP9/14 in SGs and modulate its Alu-RNA binding activity.

Another interesting noncanonical function that could be targeted is the interaction of nucleolin with the heterodimer SRP68/72 for cancer therapies and autoimmune diseases. Further studies will be able to reveal the nature of their interaction and how are these interactions regulated, providing a starting point for drug design.

Targeting SRP72 post-translational modification could be used for the treatment of patients with autoimmune diseases and/or to prevent some envelop virus replication. Future work is warranted to identify the caspase responsible for cleaving SRP72 and the kinase and phosphatase that regulate SRP72 levels.

SRP components could also be targeted to develop anti-viral therapies. For instance, considering the implication of some SRP components in HIV-1 infection, their targeting could be investigated for new antiretroviral therapies. Further research is needed to define better the role of the 7SL RNA and its fragments in retrovirus assembly. Functional and structural studies are warranted to explore the interaction of SRP components, such as SRP9, SRP14 and SRP54, with HIV-1 proteins, such as Gag and Rev. Some of the interactions between the SRP components and different viruses are expected to be similar which will facilitate the drug development process.

## Conclusion

The SRP components are extensively involved in a large range of cellular processes other than its original role in protein translocation, underlying a dynamic plasticity of SRP RNPs in cells. Considering the complex new scenario of SRP and its noncanonical functions, this RNP can no longer be categorized solely under its own canonical function.

Future studies focusing on noncanonical functions of SRP components are urgently needed to elucidate the role of SRP in signaling cascades that influence vital cellular processes and cancer development. For instance, the identification of novel interacting partners and coactivators are warranted to illustrate the role of SRP in these pathways.

Characterizing the spatial-temporal dynamic composition of SRP RNPs during different cell processes and diseases could reveal new functions for SRP components that have not been explored yet. While unveiling the proteome and transcriptome associated with SRP could identify new cellular partners and functions under physiological cell processes.

Whether therapies will be tailored toward the 7SL RNA that has been extensively linked to cancer and malignancy, or to block some specific interactions of the SRP proteins with other cellular partners, there are more noncanonical functions to be uncovered and explored yet. A new unexplored SRP world is emerging, with several “SRPrizes” which play a significant role in diseases and dynamic cellular processes.
